# Supplementary material for: Spatializing stigma-power: Mental health impacts of spatial stigma in a legally-excluded settlement in Mumbai, India
Source: PLOS Glob Public Health. 2023 Jul 20;3(7):e0001026. doi: 10.1371/journal.pgph.0001026 (PMC10358916; doi:10.1371/journal.pgph.0001026)
Supplement: Attachment — Submitted filename: Dev et al_PloS Response to Reviewers.docx [file pgph.0001026.s001.docx]

**RESPONSE TO JOURNAL REQUIREMENTS AND REVIEWER COMMENTS**

**JOURNAL REQUIREMENTS:**

1. In the ethics statement in the Methods, you have specified that verbal consent was obtained. Please provide additional details regarding how this consent was documented and witnessed, and state whether this was approved by the IRB.

**Author’s Response:** We have expanded on how the consent was documented and witnesses (lines 485-492). The procedure to obtain consent as well as verbal consent forms were approved by PUKAR’s IRB.

2. Please amend your detailed Financial Disclosure statement. This is published with the article. It must therefore be completed in full sentences and contain the exact wording you wish to be published.
   a. State the initials, alongside each funding source, of each author to receive each grant.

**Author’s Response:** Information added.

b. State what role the funders took in the study. If the funders had no role in your study, please state: “The funders had no role in study design, data collection and analysis, decision to publish, or preparation of the manuscript.”

**Author’s Response:** Added to the statement.

c. If any authors received a salary from any of your funders, please state which authors and which funders. If you did not receive any funding for this study, please simply state: “The authors received no specific funding for this work.

**Author’s Response:** Information added.

3. Please provide separate figure files in .tif or .eps format only and remove any figures embedded in your manuscript file. Please also ensure that all files are under our size limit of 10MB.

For more information about figure files please see our guidelines:

[https://journals.plos.org/globalpublichealth/s/figures](https://nam12.safelinks.protection.outlook.com/?url=https%3A%2F%2Fjournals.plos.org%2Fglobalpublichealth%2Fs%2Ffigures&data=05%7C01%7CL.Weinstein%40northeastern.edu%7C392e86b51c6741876cc308dada177897%7Ca8eec281aaa34daeac9b9a398b9215e7%7C0%7C0%7C638062091632971209%7CUnknown%7CTWFpbGZsb3d8eyJWIjoiMC4wLjAwMDAiLCJQIjoiV2luMzIiLCJBTiI6Ik1haWwiLCJXVCI6Mn0%3D%7C3000%7C%7C%7C&sdata=fn4sfrajX6WB3wEJlsH%2BLZwDLc5v5HSuyRX2wUnmkl0%3D&reserved=0) 
[https://journals.plos.org/globalpublichealth/s/figures#loc-file-requirements](https://nam12.safelinks.protection.outlook.com/?url=https%3A%2F%2Fjournals.plos.org%2Fglobalpublichealth%2Fs%2Ffigures%23loc-file-requirements&data=05%7C01%7CL.Weinstein%40northeastern.edu%7C392e86b51c6741876cc308dada177897%7Ca8eec281aaa34daeac9b9a398b9215e7%7C0%7C0%7C638062091632971209%7CUnknown%7CTWFpbGZsb3d8eyJWIjoiMC4wLjAwMDAiLCJQIjoiV2luMzIiLCJBTiI6Ik1haWwiLCJXVCI6Mn0%3D%7C3000%7C%7C%7C&sdata=nsHEu9ygXUEAZj6FR%2FU%2BTt4O%2B8FxKxxzKbBAnJ5fcwk%3D&reserved=0)

**Author’s Response:** Separate figure files in .tif format have been uploaded, and the corresponding figured have been removed from the manuscript file.

4. We notice that your supplementary table is included in the manuscript file. Please remove them and upload them with the file type 'Supporting Information'. Please ensure that each Supporting Information file has a legend listed in the manuscript after the references list.

**Author’s Response:** Based on the revisions, we have now embedded this table within our manuscript (see, Table 2, Page 9-10) instead of making this supplemental.

5. In the online submission form, you indicated that "Given confidentiality and privacy concerns, de-identified qualitative data will be available from authors upon request". All PLOS journals now require all data underlying the findings described in their manuscript to be freely available to other researchers, either 1. In a public repository, 2. Within the manuscript itself, or 3. Uploaded as supplementary information.
   This policy applies to all data except where public deposition would breach compliance with the protocol approved by your research ethics board. If your data cannot be made publicly available for ethical or legal reasons (e.g., public availability would compromise patient privacy), please explain your reasons by return email and your exemption request will be escalated to the editor for approval. Your exemption request will be handled independently and will not hold up the peer review process, but will need to be resolved should your manuscript be accepted for publication. One of the Editorial team will then be in touch if there are any issues.

**Author’s Response:** These data were collected through in-depth interviews with individuals living in marginalized urban slum communities and audio recordings were transcribed into detailed narrative transcripts. We only present de-identified data in the manuscript, with study participants’ names anonymized using pseudonyms, and we were very careful to provide limited information from the interview transcripts, such that there is no risk that study participants could be identified.

Our concern with public deposition of the full narrative information is that, even with de-identified transcripts, these extended interview data provide enough specific information that there is a risk that study participants could be identified. As such, release of this narrative data could result in the risk of ethical breaches, particularly breaches of confidentiality. Our informed consent forms specifically noted that only excerpts of information would be provided in published manuscripts or other research outputs, such that individuals would not be identifiable.

6. Images 1 and 2: please (a) provide a direct link to the base layer of the map (i.e., the country or region border shape) and ensure this is also included in the figure legend; and (b) provide a link to the terms of use / license information for the base layer image or shapefile. We cannot publish proprietary or copyrighted maps (e.g. Google Maps, Mapquest) and the terms of use for your map base layer must be compatible with our CC-BY 4.0 license.

Note: if you created the map in a software program like R or ArcGIS, please locate and indicate the source of the basemap shapefile onto which data has been plotted.

If your map was obtained from a copyrighted source please amend the figure so that the base map used is from an openly available source. Alternatively, please provide explicit written permission from the copyright holder granting you the right to publish the material under our CC-BY 4.0 license.

Please note that the following CC BY licenses are compatible with PLOS license: CC BY 4.0, CC BY 2.0 and CC BY 3.0, meanwhile such licenses as CC BY-ND 3.0 and others are not compatible due to additional restrictions.

If you are unsure whether you can use a map or not, please do reach out and we will be able to help you. The following websites are good examples of where you can source open access or public domain maps: 
* U.S. Geological Survey (USGS) - All maps are in the public domain. ([http://www.usgs.gov](https://nam12.safelinks.protection.outlook.com/?url=http%3A%2F%2Fwww.usgs.gov%2F&data=05%7C01%7CL.Weinstein%40northeastern.edu%7C392e86b51c6741876cc308dada177897%7Ca8eec281aaa34daeac9b9a398b9215e7%7C0%7C0%7C638062091632971209%7CUnknown%7CTWFpbGZsb3d8eyJWIjoiMC4wLjAwMDAiLCJQIjoiV2luMzIiLCJBTiI6Ik1haWwiLCJXVCI6Mn0%3D%7C3000%7C%7C%7C&sdata=zzgocF9giUrlBNDQKFL1M6FoY1GMn02wLrdc4HFL1uc%3D&reserved=0)) 
* PlaniGlobe - All maps are published under a Creative Commons license so please cite “PlaniGlobe, [http://www.planiglobe.com](https://nam12.safelinks.protection.outlook.com/?url=http%3A%2F%2Fwww.planiglobe.com%2F&data=05%7C01%7CL.Weinstein%40northeastern.edu%7C392e86b51c6741876cc308dada177897%7Ca8eec281aaa34daeac9b9a398b9215e7%7C0%7C0%7C638062091632971209%7CUnknown%7CTWFpbGZsb3d8eyJWIjoiMC4wLjAwMDAiLCJQIjoiV2luMzIiLCJBTiI6Ik1haWwiLCJXVCI6Mn0%3D%7C3000%7C%7C%7C&sdata=ODir9i4WRh%2FNG01UA1qmyXiUyHbkI2ybqdP5frR9ezU%3D&reserved=0), CC BY 2.0” in the image credit after the caption. ([http://www.planiglobe.com/?lang=enl](https://nam12.safelinks.protection.outlook.com/?url=http%3A%2F%2Fwww.planiglobe.com%2F%3Flang%3Denl&data=05%7C01%7CL.Weinstein%40northeastern.edu%7C392e86b51c6741876cc308dada177897%7Ca8eec281aaa34daeac9b9a398b9215e7%7C0%7C0%7C638062091632971209%7CUnknown%7CTWFpbGZsb3d8eyJWIjoiMC4wLjAwMDAiLCJQIjoiV2luMzIiLCJBTiI6Ik1haWwiLCJXVCI6Mn0%3D%7C3000%7C%7C%7C&sdata=HTRMGAjaz0TcueR%2BcDee4v66rriSsAwVKnyP5Gy78o4%3D&reserved=0)) 
* Natural Earth - All maps are public domain. ([http://www.naturalearthdata.com/about/terms-of-use/](https://nam12.safelinks.protection.outlook.com/?url=http%3A%2F%2Fwww.naturalearthdata.com%2Fabout%2Fterms-of-use%2F&data=05%7C01%7CL.Weinstein%40northeastern.edu%7C392e86b51c6741876cc308dada177897%7Ca8eec281aaa34daeac9b9a398b9215e7%7C0%7C0%7C638062091632971209%7CUnknown%7CTWFpbGZsb3d8eyJWIjoiMC4wLjAwMDAiLCJQIjoiV2luMzIiLCJBTiI6Ik1haWwiLCJXVCI6Mn0%3D%7C3000%7C%7C%7C&sdata=9qUhcJhGgvC1afWqZs9ZSjudSK6vE%2BhNnmBWrt0CE54%3D&reserved=0))

**Author’s Response:** We have reached out to the editorial office for further help with addressing this requirement.

**REVIEWERS' COMMENTS:**

1. Thanks – this is a fascinating and important paper and the abstract is brisk and clear.

**Author’s Response:** We deeply appreciate reviewer’s support for the manuscript.

2. I think for a global public health paper the paper could generally lean into a more concise and simple phraseology – the social science influence is evident in lengthy sentences and poly syllabic vocabulary which in some instances could be phrased more simply and accessibly. This is important in a global health journal where English is not a first language for many readers. For example a sentence like this takes quite slow and careful reading to elicit the intended meaning: Most importantly, we found that structural factors independently influenced disinvestment in infrastructural resources for KB as a form of exercise of stigma power to achieve the goals of eviction and clearing up the land.

**Author’s Response:** We thank the reviewer for this crucial feedback. We have now revised the entire manuscript with readability for a diverse audience in mind.

3. Researcher stance and authorship– can you please clarify the intersectional identity of authors and how they contributed. In particular, as the first three and two senior authors are affiliated with a HIC institutions and not with the Mumbai based research organisation, how does this paper contribute to reversing of the gross inequities in authorship related to LMIC and HIC residents of authors, which have been perpetuated for many decades ie. where the people with local knowledge and residence are assigned low prestige authorship positions. The asymmetry in authorship is stark and mirrors the power and privilege of authorship for high income country residing authors, an important concern, especially in a paper like this where the focus is on local knowledge/ context/ location and power. A discussion of authorship stance would also be important to include in the methods section to acknowledge how intersectional identity influences research design and reporting.

**Author’s Response:**

We thank the reviewer for pointing out the missing researcher positionality statement, as well as motivating us to address the potential power differentials in collaborative global health research. We have added a section to our methods section (lines 570-605) that addresses this comment. Particularly, we have documented geographical locations, education and training, and career status of each of the author. In addition, we have also explained contributions of each author. Of note, the current authorship sequence reflects the secondary nature of this study whereby data collected by some of the authors (TS, KS, SS, APD, and RS; 4^th^ through 7^th^ and 9^th^) were re-analyzed by applying a new theoretical framework. The first author led the study conceptualization, data analyses, interpretation, and manuscript writing. The second and third authors were integral part of the data analysis process and interpretation of findings. Senior authors (LW and RS) over-sought the entire process and with the help of all the authors, made revisions to the manuscript. In deciding the authorship for this manuscript, we have prioritized analytical and theoretical contributions rather than data collection efforts.

**Introduction**

4. Literature is well summarised and the research gap clearly identified. Given the Dhaka study on spatial sitgma is reported as the only one in South Asia it would be useful to get a one sentence summary of that.

**Author’s Response:**

We thank the reviewer for the encouraging feedback on the literature review section. In lines 203-206, we have added a summary of the study that was conducted in Dhaka.

5. Can you give further data or evidence showing that this informal urban setting or residents in slums generally have higher rates of CMDs than other settings where people with low income reside in India/ Mumbai?

**Author’s Response:**

We have added such evidence from slums across the world, as well as findings from a previous study in Mumbai that reported CMD prevalence of 23%, which is the highest rate described in any population-based study in India. (see, lines 153-154 and lines 168-185).

6. Given the centrality of the Pearce framework on spatial stigma which it seems was developed in a high income context, it would be useful to consider what contextual factors are at play and different in the setting you describe - eg in India/ South Asia, especially as ‘slums’ have more legally contested locations which adds another layer of structural disadvantage.

**Author’s Response:**

We thank the reviewer for pointing out this gap. In the conclusions section, we have made our contributions to the field more explicit, especially with respect to the application of Pearce’s framework to a new setting and the attributes of the context-specific framework that emerged from the findings. (see, lines 951-976).

7. Aims - I think this is your statement of aim – can you say more simply? In this paper, we draw on the valuable, but still limited research on spatial stigma and health to investigate the extent to which stigmatized perceptions of a neighborhood may help explain the high prevalence of probable common mental health disorders (CMD) in a non notified slum (i.e., settlement lacking legal recognition by the government) in Mumbai, India. Or is this other statement also apparently summarising the study aim : In the analysis that follows, we use this integrated conceptual framework as a starting point for interrogating pathways between spatial stigma and mental health in a slum settlement in Mumbai, India, that has been previously shown to have a very high prevalence of CMDs.(25) Of note, this is also an inquiry into the applicability of Pearce’s (38) conceptual framework in the slum context in India, and the inter-relationships between these pathways (Pearce (38) acknowledged that these pathways are non-mutually exclusive but did not articulate the relationships between them). It would be preferable to state study objectives in one place and state things more directly EG We will use the Pearce framework to interrogate pathways between spatial stigma and poor mental health and also examine how applicable and relevant the Pearce framework is in a slum setting in India.

**Author’s Response:** We have made the recommended change to the introduction section in lines 143-145: *“In this paper, we investigate the pathways through which spatial stigma impacts mental health of residents in a non-notified slum (i.e., settlement lacking legal recognition by the government) in Mumbai, India.”*

We later (towards the end of the literature review, before we discuss the structural origins of spatial stigma in KB) re-state the aim for the convenience of the readers and have simplified the language in lines 267-272: *“In the analysis that follows, we use this conceptual framework for interrogating pathways between spatial stigma and mental health in a slum settlement in Mumbai, India, that has been previously shown to have a very high prevalence of CMDs.(25) We also examine the applicability and relevance of Pearce’s (38) conceptual framework in the slum context in India, and the inter-relationships between these pathways. We begin this investigation by providing an overview of the history of our research setting, below.”*

8. History of study setting – wonderful to see this written with clear pointing to colonisation and historical health determinants, refreshing and hopefully sets the standard in political economy of health and place.

**Author’s Response:** We deeply appreciate the reviewer’s enthusiasm towards this section.

9. Appreciate the rationale for relevance of data collected over a decade ago.

**Author’s Response:** We thank the reviewer for this comment.

10. Table 1 – notable is the absence of information on caste identity as a major health determinant in India. Did you gather this information and can it be added? If not can you please comment on caste and how it may have interacted in findings of this study. Also, perhaps you can give a small rationale for your definition of North and South Indians and relevance of those identities vs Maharashtrians.

**Author’s Response:** Given the sensitivity around discussions on caste, data on this topic was not collected. However, in lines 1121-1125, we do acknowledge and discuss “the intersection of multiple ethno-religious ethnicities and caste status that are relevant in this locality” in the context of future research directions.

In addition, we have now included a footnote to Table 1 (page 9) that defines North and South Indians, and gives relevance to the characterization of participants based on their North Indian, South India, or Maharashtrian ethnicity: *“”North Indians” are usually natives or descendants of natives of the northern states of Delhi, Jammu and Kashmir, Himachal Pradesh, Punjab, Uttaranchal, and Haryana. “South Indians” are natives or descendants of natives of the southern states of Tamil Nadu, Karnataka, Kerala, Andhra Pradesh, and Telangana. We characterize participants based on these three ethnic identities given the history of ethnic scapegoating against North and South Indians in Maharashtra, as well as the ethno-nationalist movement promoting “Maharashtrians” or natives/descendants of natives of Maharashtra (as described in the section “Structural Origins of Spatial Stigma in Kaula Bandar”).”*

**Methods:**

11. See comment earlier about stance of authors.

**Author’s Response:** We have now included a paragraph in the methods section to address researcher positionality and authorship (see lines 614-649).

**Results:**

12. Nice to get social identity of people who participate in verbatim quotes – thank you.

**Author’s Response:** We thank the reviewer for the support and appreciation.

13. Apart from the data around self stigma, the authors present few results linking mental distress and structural determinants directly. I think it is reasonable to assume that the textured descriptions of social stigma and how it operates lead to mental distress but wondering if you have any data that draws this connection more explicitly.

**Author’s Response:** We thank the reviewer for highlighting this aspect. We would like to underscore the connections we draw between spatial stigma and “tension” which is a local idiom of distress in India for depression, anxiety, and psychological distress. See lines 661-663, and 726-745.

14. This study presented a largely deficit focused summary of the mental health stigma experiences and of discrimination and exclusion linked to residence in this slum. I appreciated the small section in results which identified a positive neighbourhood identity of community cohesion and lack of communitarian violence during the early 90’s. There is a risk in presenting such a paper that it further entrenches negative assessments of this and similar slums with a largely negative picture. I wonder if you have any further data that can also examine and nuance your data by also describing community assets and examples of mental health resilience or strength that may also be linked to this spatial location. This would add some nuance and I believe would not take away from the over-arching study question.

**Author’s Response:** We again appreciate this thoughtful comment by the reviewer. In our attempt to highlight stigmatization of KB, we understand and have actively tried to address the risks we might pose to further stigmatize this community. In particular, even when community residents did engage in lateral denigration of other community members, we were careful to contextualize these statements by pointing out that such statements were often not aligned with the underlying structural causes of problems in the community. For example, on page 16, although some residents blamed solid waste challenges on other residents, we point out that the underlying cause of these solid waste challenges is actually government neglect.

In the manuscript, we have presented all the robust evidence that supported residents’ attempt to counter spatial stigma, and we have now added to the Discussion to point out that we occasionally identify counter-narratives of solidarity that result as a response to spatial stigma, similar to the Fattah and Walters study in Dhaka, Bangladesh. At the same time, while residents discussed community assets, we found little evidence around resilience given the multiple layers of systemic discrimination the residents face. For example, even when participants recounted narratives of community cohesion, they talked about how it was ultimately negatively impacted by community’s infrastructural deficits.
