# Supplementary material for: Spatializing stigma-power: Mental health impacts of spatial stigma in a legally-excluded settlement in Mumbai, India
Source: PLOS Glob Public Health. 2023 Jul 20;3(7):e0001026. doi: 10.1371/journal.pgph.0001026 (PMC10358916; doi:10.1371/journal.pgph.0001026)
Supplement: Attachment — Submitted filename: Dev et al_PloS R2 Response.docx [file pgph.0001026.s002.docx]

**RESPONSE TO JOURNAL REQUIREMENTS AND REVIEWER COMMENTS**

**JOURNAL REQUIREMENTS:**

1. In the ethics statement in the Methods, you have specified that verbal consent was obtained. Please provide additional details regarding how this consent was documented and witnessed, and state whether this was approved by the IRB.

**Author’s Response:** We have expanded on how the consent was documented and witnesses (lines 291-302). The procedure to obtain consent as well as verbal consent forms were approved by PUKAR’s IRB.

2. Please review your reference list to ensure that it is complete and correct. If you have cited papers that have been retracted, please include the rationale for doing so in the manuscript text, or remove these references and replace them with relevant current references. Any changes to the reference list should be mentioned in the rebuttal letter that accompanies your revised manuscript. If you need to cite a retracted article, indicate the article’s retracted status in the References list and also include a citation and full reference for the retraction notice.

**Author’s Response:** We have reviewed the reference list to make sure that it is complete and correct.

**REVIEW COMMENTS TO THE AUTHOR:**
**Reviewer #2:**

1. The authors need to further explain how their findings reflects the socio-ecological framework they have used in this study. Also the authors must show the link between the socio-ecological framework and Pearce Conceptual Model in relation to the five pathways identified in the study.

**Author’s Response:** We appreciate the reviewer’s comment and for the opportunity to clarify this. In the discussion section (lines 839-851), we have now explained how the findings reflect the socio-ecological framework. In addition, in the methods section (lines 357-364), we have explained the link between the socio-ecological framework and the pathways identified within Pearce’s conceptual model. Specifically, we included the following text to describe how our collection of qualitative data using a socio-ecological framework is what enabled us to map these findings onto the multi-level processes leading to spatial stigma in Pearce’s framework:

“*Pearce’s framework especially lends itself to analysis of the data guided by the socio-ecological framework given that Pearce’s pathways assume a multi-level approach to spatial stigma ranging from structural origins of spatial stigma and resultant disinvestment at the macro-level, discrimination and weakened solidarity at the community and inter-personal levels, and internalization at the individual level. As such, our use of the socio-ecological framework in our FGD and interview guides to explore challenges faced by participants at the individual, family, and community levels is what enabled us to map findings onto the multi-level processes that shape spatial stigma in Pearce’s framework.”*

2. The authors should add a map before the Methods section to depict the social process that has led to some form of change in the social environment.

**Author’s Response:** We greatly appreciate this feedback. We believe that Figure 1 and Figure 2 already present an initial model and a modified model, based on our findings, that attempt to map social processes that lead to spatial stigma and its subsequent adverse impact on social life and health outcomes. We currently face uncertainty regarding the creation of an additional figure that would delineate social processes, considering that the existing figures within the manuscript comprehensively depict these crucial processes. We are also worried that an additional figure runs the risk of confusing the reader. We have taken the reviewers’ feedback on articulating social processes very seriously as described in our response to point #3 below, and we have carefully reviewed the manuscript to ensure that social processes leading to spatial stigma and then poor mental health are articulated throughout the Results.

3. The social processes of spatial stigma should be discussed. This is because; spatial stigma is a new social construction of reality within the domain of public health research.

**Author’s Response:** We thank the reviewer for the feedback. In our results and discussion section, we discuss two social processes of spatial stigma and its relevance for public mental health. First, we discuss Stigma Power that draws attention to the enactment of stigma through the exploitation, exclusion, or exertion of control over stigmatized individuals (lines 186-187, 421-423, 443-529). Disinvestment in KB reflects the exercise of stigma power to achieve the goals of eviction and clearing up the land on which KB sits. These actions contribute to mental health challenges by hindering social cohesion and limiting access to essential resources. We elaborate on this process in lines 447-453: “*Stigma power was actively utilized by public authorities in the form of social disinvestment and collective punishment, for example by cutting non-formal electricity wires or raiding and removing water pumps to cut off the community’s water supply. This further marginalized the community by impacting social structures, for example, by enabling the police and other government officials to extract bribes for any basic services that are accessed by residents, since any such access is technically* *illegal.”* And in lines 495-502: “*Ahmed’s account of access to water and electricity in KB clearly demonstrates the cyclic nature of how structural and infrastructural factors are a cause and consequence of the stigma attached to KB. In the absence of legal ways of accessing these fundamental needs, some KB residents obtain water and electricity through informal means, which can be framed through the stigmatizing language of “illegality”. This reveals that when the governmental inaction denies the residents legal ways to access water and electricity, stigma power can further amplify stigma. When someone filed a complaint against these residents, their informal connections were taken away, revealing how spatial stigma can lead to further contribute to material marginalization.*”

Second, we discuss the process of lateral denigration whereby residents distanced themselves from “other” residents and claimed that the stigma attached to KB applies to their neighbors but not themselves (lines 196-201). This perpetuates the marginalization and discrimination experienced by specific individuals or groups. By engaging in lateral denigration, residents inadvertently contribute to the broader social construction of spatial stigma and its detrimental effects on mental health, community relations, and overall well-being (lines 600-710). Specifically, in lines 609-616, we explain, “*Some specific examples of participants displacing stigma onto their neighbors included perceptions that other KB residents often fight with each other to obtain access to basic services, generalization of individual instances of crime (e.g., robbery) as being indicative that the community more broadly is morally compromised, and concerns that other ethnic groups within the community are not “good people.” In some cases, concerns about specific ethnic groups in the community reflected that fact that populations who had been in the community for a longer time (e.g., Tamilians) had secured relative control over informal selling of water and electricity*.” Our findings highlight he importance of addressing spatial stigma as a social determinant of health to foster individuals’ mental health and well-being.

4. The sub-section “Conclusion” should be replaced with “Discussion/Conclusion” or this should be separated.

**Author’s Response:** We appreciate this valuable suggestion. We have now replaced “Conclusion” with the heading “Discussion”, and have added a sub-section titled “Conclusion” towards the end of the Discussion section.

5. The authors should consider adding “India” at the end of their title after “Mumbai”.

**Author’s Response:** We have now added “India” at the end of our manuscript title.

6. The manuscript should be double-checked prior to publication to address any form of spelling mistakes on error.

**Author’s Response:** We have proof-read the manuscript and addressed spelling errors.

7. The study is quite interesting and its replication in other settings will further assert the concepts of “Stigma Power” and Spatial Stigma.

**Author’s Response:** We deeply appreciate the reviewer’s note on the value this study brings to the field. We hope that this work inspires future scholarship on the link between spatial stigma and health and, more importantly, interventional work to ameliorate the underlying processes that lead to spatial stigma.
